# A Contribution towards Sustainable Development in the Amazon Based on a Socioeconomic and Environmental Analysis of Visceral Leishmaniasis in the State of Pará, Brazil

**DOI:** 10.3390/tropicalmed9030066

**Published:** 2024-03-21

**Authors:** Claudia do Socorro Carvalho Miranda, Bruna Costa de Souza, Eric Renato Lima Figueiredo, João Simão de Melo Neto, Hilton Pereira da Silva, Marcos Valerio Santos da Silva, Sérgio Luiz Althoff, Tainara Carvalho Garcia Miranda Filgueiras, Debora do Socorro Carvalho Miranda, Nelson Veiga Gonçalves

**Affiliations:** 1 Laboratory of Epidemiology and Geoprocessing of Amazon, University of the State of Pará (UEPA), Belém 66050-540, Brazil; bruna.souza@uepa.br (B.C.d.S.); leonardo.miranda@icen.ufpa.br (D.d.S.C.M.); nelsonveiga@uepa.br (N.V.G.); 2 Programa de Pós-Graduação em Saúde Coletiva na Amazônia, Federal University of Pará (UFPA), Belém 66075-110, Brazil; eric.figueiredo@ics.ufpa.br (E.R.L.F.); jsmeloneto@ufpa.br (J.S.d.M.N.); hdasilva@ufpa.br (H.P.d.S.); marcossilva@ufpa.br (M.V.S.d.S.); 3 Programa de Pós-Graduação em Inteligência Territorial e Sustentabilidade, Centro Universitário do Pará (Cesupa), Belém 66060-230, Brazil; 4 Center for Advanced Multidisciplinary Studies, University of Brasília, Brasília 70910-900, Brazil; 5 Animal Biology Laboratory, Natural Sciences Department, Blumenau Regional University (FURB), Blumenau 89012-078, Brazil; althoff@furb.br; 6 Programa de Pós-Graduação em Direito, Federal University of Pará (UFPA), Belém 66075-110, Brazil; tainara.filgueiras@ifch.ufpa.br; 7 Cyberspace Institute, Federal Rural University of Amazon (UFRA), Belém 66077-830, Brazil; 8 Programa de Pós-Graduação em Biologia Parasitária na Amazônia, University of the State of Pará (UEPA), Belém 66050-540, Brazil

**Keywords:** visceral leishmaniasis, epidemiology, environment, spatial analysis

## Abstract

Human Visceral Leishmaniasis is an endemic public health problem in the Amazon. This article analyzed the spatial distribution of this disease and its relationship with socioeconomic, environmental and public health policy variables in four mesoregions of the state of Pará, from 2011 to 2022. This ecological study used secondary data obtained from official Brazilian agencies. Spatial analysis was performed using the Flow, Kernel and Global Moran bivariate techniques expressed in thematic maps. In the mesoregions studied, 2685 cases of the disease were confirmed, with the highest number of cases in Southeast Pará state. The epidemiological profile followed the national pattern of occurrence of the disease, with a higher number of cases in children below school age. Spatial dependence was observed between the prevalence of the disease and socio-economic indicators. The most intense movement of patients was towards the Belém Metropolitan mesoregion. The disease showed an inhomogeneous pattern of distribution of cases, with a direct relationship between areas with cases and deforestation associated with different anthropic activities. There is a socio-environmental production of the disease that goes beyond the border limits of the mesoregions, and its establishment is related to the unsustainable development model implemented in the region.

## 1. Introduction

Human Visceral Leishmaniasis (HVL) is a chronic, systemic zoonosis caused by different species of protozoa of the genus *Leishmania* transmitted to hosts by the bite of the infected female phlebotomine during the blood meal. In urban environments, its main reservoir is the domestic dog (*Canis familiaris*, Lineu, 1758), while in the wild environments of South America, the reservoirs are foxes (*Lycalopex vetulus*, Lund, 1842 and *Cerdocyon thous*, Lineu, 1766) and white-eared opossums (*Didelphis albiventris*, Lund, 1840) [1,2].

HVL is widely distributed throughout the world and is present on all continents and endemic in 80 countries. The World Health Organization (WHO) estimates the occurrence of 50,000 to 90,000 new cases per year, with approximately 2 billion people worldwide living in areas at risk of transmission of the disease [3]. The highest number of notifications occurs in Brazil, East Africa and India. In the Americas, this disease has already been reported in 13 countries. Brazil is responsible for 93% of cases of the disease [4].

According to data from the Ministry of Health (MS), approximately 3500 cases of HVL are reported annually in Brazil, with an incidence rate of 2.0 cases/100,000 inhabitants [2,5]. Between 2011 and 2021, the lethality of the disease increased gradually from 4.1% to 12.8% [6]. Autochthony of the disease has been confirmed in 24 states distributed across the five Brazilian regions [2,5,7]. In this context, the Ministry of Health developed the Visceral Leishmaniasis Control Program (PCLV) with the aim of reducing lethality rates and the degree of morbidity of the disease, as well as reducing the risks of transmission through the control of reservoirs and vectors. Because it presents a public health risk, this disease must be reported to health authorities so that they can adopt intervention measures with a view to preventing, monitoring and controlling the disease [8,9].

In Brazil, especially in the Amazon, the importance of HVL lies in its high incidence, high lethality, wide distribution and the magnitude of its morbidity and mortality, as well as the impact it has on the Unified Health System (SUS) and its socio-economic implications [2,5,10,11,12]. This vector-borne anthropozoonosis mainly affects vulnerable populations and is associated with serious socio-environmental problems [13,14,15,16]. However, given the epidemiological pattern of its distribution and risk factors, the lack of knowledge about the regions and populations most susceptible to developing the disease is a problem for drawing up public monitoring and surveillance policies [3,4,8]. This is especially the case in areas considered endemic, where the number of people exposed or infected may be much higher than the number of cases detected, which underscores the ongoing need to expand monitoring and confirmation of the disease.

Pará is the second largest state in the Amazon region and the administrative mesoregions into which its territory was divided have distinct historical, socioeconomic and environmental characteristics. They are generally determined by the exploitative model of occupation and land use implemented in the region [17,18,19,20,21], which has led to the disruption of natural systems with a high rate of deforestation and environmental degradation that exposes the population to risk factors for the disease in this part of northern Brazil [13,14,15,18,19]. In the period from 2020 to 2022, the state had thirteen municipalities classified as being at intense risk of transmission and six at high risk, which highlights the difficulties in dealing with this disease [22,23].

With that in mind, geotechnology has been widely used in health studies to assist in producing information on infectious and parasitic diseases, enabling them to be studied by integrating an unlimited amount of cartographic information [24,25,26,27]. These tools make it possible to characterize the geographical distribution of the disease and its risk factors and aid in programs to address it in the different realities of the Amazon. In view of the above, this study aimed to analyze the relationship between the spatial distribution of HVL and environmental, socioeconomic and public health policy variables in four mesoregions of the state of Pará, from 2011 to 2022.

## 2. Materials and Methods

**Figure 1:**
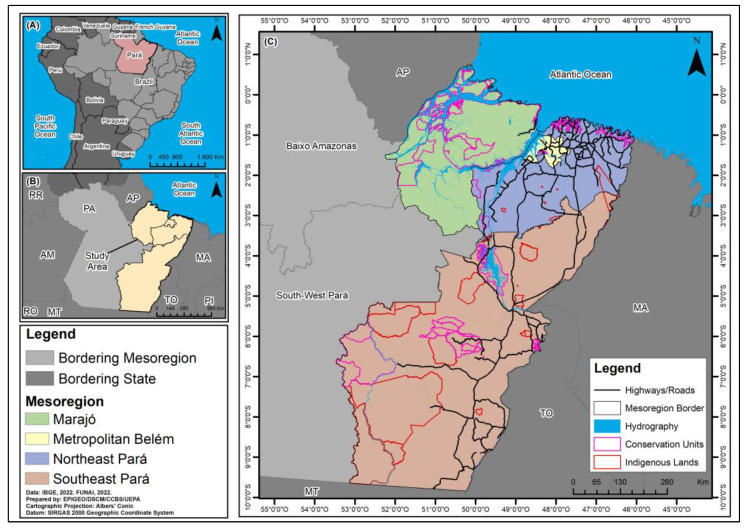
Spatial location of Pará [(**A**), red], study area [(**B**), beige], and map of the Marajó, Metropolitan Belém, Northeast Pará and Southeast Pará mesoregions (**C**), Pará, Brazil.

An ecological study was carried out in all laboratory-confirmed cases of HVL in four mesoregions of the state of Pará from 2011 to 2022. The spatial units of analysis were the mesoregions of Marajó (16 municipalities with 577,790 inhabitants), Metropolitan Belém (11 municipalities with 2,751,161 inhabitants), northeast Pará (49 municipalities with 2,023,064 inhabitants) and Southeast Pará (39 municipalities with 976,523 inhabitants) [28] (Figure 1).

Epidemiological data (gender, age group, ethnic groups, schooling, area of residence and prevalence) were obtained from the Ministry of Health Notifiable Diseases Information System. The information on geographical boundaries, population, and economic indicators (Gross Domestic Product—GDP) was sourced from the 2010 Census database provided by the Brazilian Institute of Geography and Statistics. The environmental databases related to land use and land cover (forest, forest plantation, natural formation, pasture, agriculture, non-vegetated area, urban area, mining and water) used in this work were obtained from the Land Use and Land Cover Collection 8 (2022) at the MapBiomas Collections.

The data obtained were debugged to remove inconsistencies and incompleteness and for subsequent indexing in a Geographic Database (BDGeo), using Tabwin 415 software (Brazilian Ministry of Health, Brasilia Federal District, Brazil). The confirmed cases of HVL were then georeferenced in the field and laboratory using a Global Positioning System (GPS) and then stored in BDGeo. Field trips were made to the study area to verify the veracity of the terrain. The descriptive analysis used percentage calculations and the non-parametric chi-square statistical test of equal expected proportions with a significance level of 0.05, using the Bioestat 5.4 program (Brazilian Ministry of Health, Brasilia Federal District, Brazil).

In the analysis of spatial distribution (direction and orientation), the prevalence (cases/population × 100,000) and GDP of the study mesoregions were presented on choropleth maps. The prevalence was stratified into 6 intervals (no cases, very low, low, moderate, high and very high) using the quantile technique. The flow technique was used in order to perform the demand/supply analysis of access to treatment. The municipal seats of the municipalities where patients lived and where they received treatment were identified and mapped. The flow map was constructed by calculating the distances between the municipalities of origin and destination, classified by natural breaks, with a category distribution that made it possible to stratify the distances into very close (up to 53 km), close (53 to 160 km), medium (160.1 to 277 km), far (277.1 to 390 km) and very far (390.1 to 1073 km), represented by lines with color gradients where the arrows indicate the final destination. All these analyses were carried out using ArcGIS 10.5.1 software (ESRI, Redlands, CA, USA).

The Kernel Density Estimate was used to analyze the spatial distribution of the disease in order to identify possible clusters of cases. In building the land use and cover map, the following classes were considered: forest, forest plantation, natural formation, pasture, agriculture, non-vegetated area, urban area, mining and water. The Bivariate Global Moran’s index (I) was used to assess the spatial autocorrelation between the areas with deforestation and those with cases of HVL. It was considered direct for I > 0, inverse for I < 0, and strong when indices were close to one of the defined variation limits (−1;1), with a significance of *p* < 0.05. All these analyses were carried out using the ArcGIS 10.5.1 (ESRI, Redlands, CA, USA) and GeoDa 1.22 (University of Chicago, Chicago, IL, USA) software.

Ethical aspects were ensured in this study in accordance with the Declaration of Helsinki, the Nuremberg Code and the norms of Resolution 466/12 of the National Health Council. This study received a favorable opinion, number 3.292.673, from the Research Ethics Committee of the State University of Pará.

## 3. Results

During the study period, 2685 cases of HVL were confirmed in the mesoregions studied, with Southeast Pará (1376) and Northeast Pará (1070) showing the highest numbers, followed by the Metropolitan Belém (182) and Marajó (57). An analysis of the profile of individuals diagnosed with HVL showed a higher percentage of occurrences in males, aged between 0 and 12 years, in brown-skinned people with schooling as “not applicable” (children under 3 years old). Concerning the area of residence, the Northeast Pará, Marajó and Belém Metropolitan mesoregions had the highest number of cases in rural areas, while Southeast Pará reported the highest number in urban areas, all with a level of statistical significance (Table 1). In terms of the age range variable, it was observed that of the 1526 children affected by the disease, 951 (62.32%) were aged between 0 and 3 years old.

**Figure 2:**
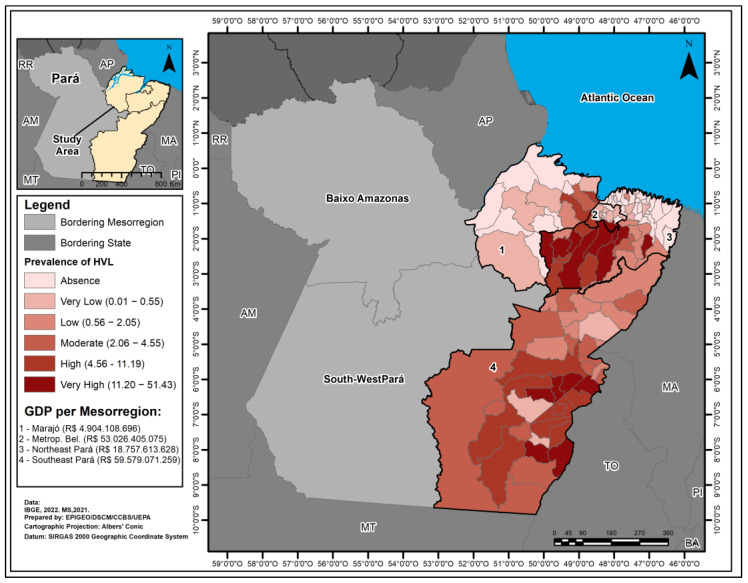
Map of the prevalence of Human Visceral Leishmaniasis and Gross Domestic Product in the Mesoregions of Marajó, Metropolitan Belém, Northeast Pará and Southeast Pará, Pará State, Brazil, from 2011 to 2022.

Analysis of the spatial distribution of HVL prevalence in the study mesoregions showed that Southeast Pará had 6 municipalities with a very high prevalence, 9 with a high prevalence and 13 with a medium prevalence. The Northeast Pará region had 9 with very high prevalence, 4 with high prevalence and 2 with medium prevalence. Metropolitan Belém had 1 with very high and 1 with high prevalence. The Marajó mesoregion had 2 with high prevalence and 1 with medium. The GDP for the mesoregions shows different values (Figure 2). 

**Figure 3:**
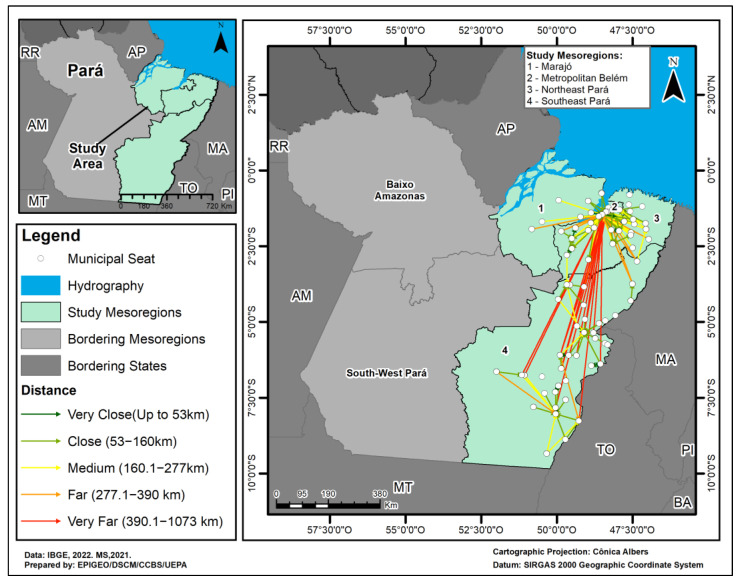
Map of the flow of patients with Human Visceral Leishmaniasis in the Metropolitan Mesoregions of Metropolitan Belém, Marajó, Northeast Pará and Southeast Pará, in the period 2011–2022.

As for the geographical distribution of the flow of HVL treatments, 202 patients living in the Northeast Pará (165 patients), Southeast Pará (27 patients) and Marajó (10 patients) mesoregions went to the municipality of Belém, in the Metropolitan Belém mesoregion. This municipality also serves as a hub for the municipalities of the same mesoregion (29 patients). Another important hub was Marabá, in the southeast of Pará, which also receives patients from the municipalities in the mesoregion. The majority of patients (202) had their treatment funded by the Unified Health Service (SUS), through Treatment Away From Home (TAFH) and the use of services in the other location, as they live more than 150 km from the place of care. The other flows are regional, with few distances covered and a small volume of patients (Figure 3).

**Figure 4:**
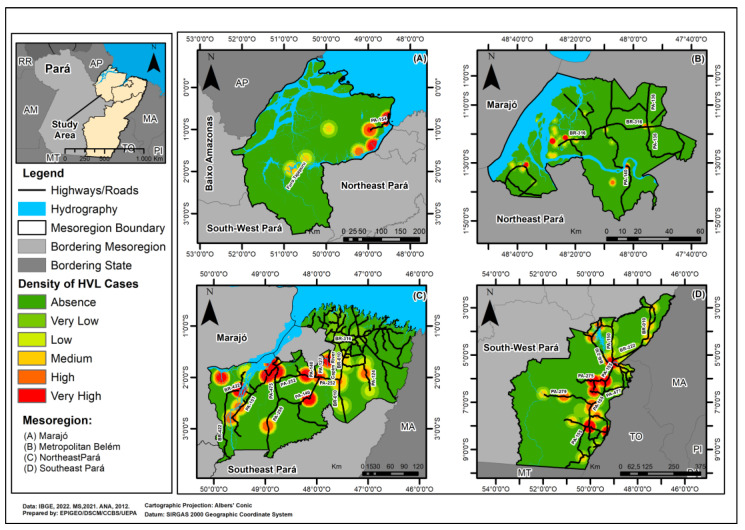
Density of Human Visceral Leishmaniasis cases in four Mesoregions of the state of Pará, Brazil, for the period 2011 to 2022: (**A**) Marajó; (**B**) Metropolitan Belém; (**C**) Northeast Pará; (**D**) Southeast Pará.

Using the Kernel technique, it was possible to identify that HVL occurrence in the mesoregions showed an inhomogeneous distribution pattern with clusters of cases along the highways and roads. A very high and high density of the disease was observed in the Northeast and Southeast mesoregions of Pará, with evidence of vectorization in their municipalities. It was also noted that the Marajó and Belém Metropolitan mesoregions had a high, medium and low density of cases (Figure 4).

A process of vectorization of the disease was identified among the municipalities that make up the northeastern and southeastern mesoregions of Pará. In northeast Pará, epidemiological corridors were identified, which involved the municipalities of Acará, Moju, Igarapé-Miri and Abaetetuba, located close to the PA-475, PA-252 and PA-151 highways. These continued on to the municipalities of Cametá and Oeiras do Pará, crossed by the BR-422 highway. At the same time, this event entered Concórdia do Pará and Tomé-Açu via the PA-140 and PA-252 state highways. Simultaneously, the flow continued to the municipality of São Domingos do Capim close to the PA-127 highway and the Capim River. In the southeastern region of Pará, this epidemiological corridor extends through an arc formed by the municipalities of Jacundá, Marabá, Curionópolis, Eldorado dos Carajás, Sapucaia, Xinguara, Rio Maria, Pau D’arco and Redenção, located on the banks of the PA-150 and PA-155, branching off to the municipality of Parauapebas, crossed by PA-275 (Figure 4).

**Figure 5:**
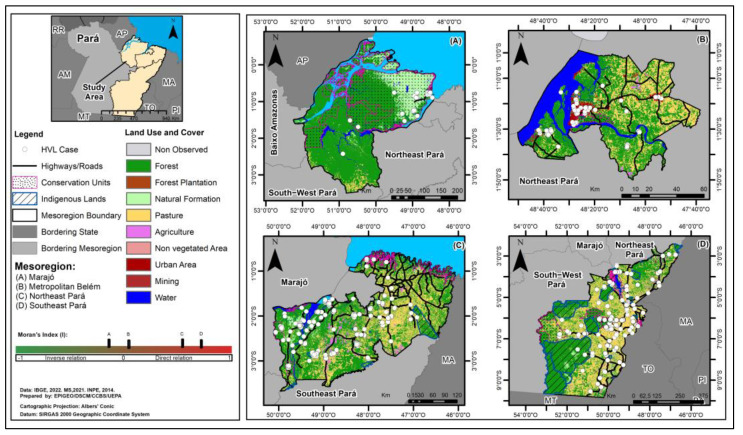
Spatial distribution of Human Visceral Leishmaniasis cases, land use and land cover and interpretation of the spatial autocorrelation of the Moran Index (I) in the four Mesoregions studied, Pará, Brazil, from 2011 to 2022: (**A**) Marajó; (**B**) Metropolitan Belém; (**C**) Northeast Pará; (**D**) Southeast Pará.

The land use and land cover map of the study mesoregions showed that in areas that had significant deforestation due to various human activities also had a greater number of cases of HVL. A very high percentage of pastures, mining and urbanization were found, as well as the presence of small secondary forest fragments and isolated forest remnants in the Belém Metropolitan, Northeast Pará and Southeast Pará mesoregions. A large number of urban areas with clusters of cases were also observed, especially in the Belém Metropolitan and Southeast Pará mesoregions. In the Marajó mesoregion, preserved forest areas were observed. Cases of HVL were also observed in and around Conservation Units (CU) and Indigenous Lands (IL) in the Northeast (IL Tembés and Anembés) and Southeast Pará mesoregions (ILs for the Assurini, Gavião Parkatejê, Suruí and Xikrin peoples) (Figure 5).

The spatial analysis of the relationship between the areas of the mesoregions that reported cases of HVL and those that showed deforestation using the Bivariate Global Moran index (I) showed significant spatial dependency relationships between these variables. These autocorrelations were direct and strong in Southeast Pará (I = 0.751) and Northeast Pará (I = 0.627), direct and weak in Metropolitan Belém (I = 0.061) and inverse and low in Marajó (I = −0.228) (Figure 5).

## 4. Discussion

The results of the research show that HVL remains a major public health challenge in the mesoregions studied due to the complex epidemiological scenario in the Pará municipalities, which have environmental and socioeconomic determinants that enable the establishment of transmission cycles for the disease, such as the hot and humid climate conducive to the development of vectors, the constant presence of reservoirs, environmental degradation, intermittent circulation of the pathogen and precarious sanitary conditions [29,30,31,32,33,34].

The epidemiological profile marked by the predominance of male and brown-skinned individuals affected by the disease indicates a recurring pattern of transmission that has been found other studies carried out in Brazilian territory [35,36,37,38]. These characteristics may be associated with behavioral, immunological, socio-demographic and environmental risk factors, including greater environmental exposure to the vectors and reservoirs of the disease due to environmental racism, male negligence with regard to caring for their own health, hormonal issues, and finally the ethnic-racial composition of the state of Pará, given that self-declared brown and black people represent approximately 80% of the residents of this territory [28,35,36,37,38].

The significant number of cases of the disease in children below school age is due to the vulnerability caused by the social and environmental inequalities that affect the majority of Amazonian populations [39,40,41], which include limited access to goods and services and the low protection of children’s health in socio-environmentally deprived areas. This age group is more susceptible to the disease because their immune system is still immature, especially in those with food and nutritional insecurity [42,43]. The majority are under 3 years old and do not yet attend school, suggesting possible transmission within and/or between homes, with these children having more contact with domestic animals, such as dogs. The combination of these factors indicates the need for interdisciplinary, integrative and multisectoral strategies based on the principles of promoting One Health in its animal, human, plant and environmental interfaces, since all these elements are closely linked and interdependent [44,45,46,47].

The rural transmission pattern of HVL in the Marajó, Belém Metropolitan and Northeast Pará mesoregions is related to the socioeconomic characteristics of population groups that have well-defined intersectionalities and social markers in the Amazon region, including subsistence farmers, extractivists and small-scale fishers. The practices of such groups are associated mainly with riverine communities, quilombolas, settlers and Indigenous people, who make up the majority of the region’s peasantry [18,31,40,42,48,49]. These populations, due to their historical origins, cultural practices and because they are inserted in the peripheral context of the economic system and its relationship with regional development, live and carry out their productive activities in areas vulnerable to the socio-environmental production of diseases.

The presence of HVL in the urban areas of all the mesoregions studied, especially in Southeastern Pará, indicates a possible process of urbanization of the disease associated with disordered population growth and the resulting socio-spatially segregated areas and the formation of structural poverty belts [32,50,51,52,53]. Such areas generally have a low economic level, a lack of environmental sanitation and precarious housing conditions, which amplify the risk factors and are conducive to the physiological and evolutionary demands of the vector, due to the presence of domestic and synanthropic animals and the precarious sanitary conditions.

This socio-spatial reorganization in the Amazon has occurred rapidly and is due to various factors, including a major large migratory flow of workers in search of jobs in development projects, the displacement of local populations for the implementation of these projects. The failure to demarcate traditionally occupied lands has favored land-grabbing processes and the expulsion of families from the countryside to the city. That has led to the formation of new peri-urban population centers located near riverbanks and residual forest areas and to a process of disordered urbanization, which contribute to the cycle of transmission of HVL [17,18,19,20,53].

Given that HVL is a disease directly associated with social vulnerability, conditioned by the socioeconomic and environmental context of human populations, the significant number of municipalities with very high and high HVL prevalence in the mesoregions that have medium and high GDP points to epidemiological scenarios that run counter to the Sustainable Development Goals (SDGs) of the 2030 Agenda, which advocates the eradication of poverty-related diseases as a global challenge, as well as the need to ensure a healthy life and promote well-being for all, at all ages [54,55].

Although the GDPs observed as medium and high in some of the mesoregions studied may be pointing to dynamic economic activities, with high growth rates, based on technological and industrial advances, this is not the reality in these territories. In addition, it is necessary to emphasize that in order to achieve the objectives of the 2030 Agenda, the form of economic accumulation linked to development of the Amazon region. This must be done with a view to guaranteeing an improvement in the living conditions of local populations, combating the relationship of primitive economic accumulation, based on the simple appropriation of territory, natural resources and speculative retention of property, in which the state participates directly with regulatory policies, in a logic of connivance or omission [56,57].

Based on this understanding, the epidemiological scenarios of HVL observed in the municipalities of Parauapebas, Canaã dos Carajás and Marabá, which have the highest GDPs in the state of Pará and are concomitantly endemic areas indicate that priority should be given in the territory of Pará to the implementation of public policies that seek to realize the basic rights of individuals recommended by the Federal Constitution of 1988. There is also a need for reviewing the theoretical-methodological and conceptual scope of the Visceral Leishmaniasis Surveillance and Control Program [8,9,32]. In this sense, in order to achieve its objectives, the program must present and apply differentiated actions adapted to each of these realities, taking into account the specificities of the mesoregions and their municipalities, allowing for adequate, rational and feasible planning.

The spatial analysis using the flow map showed a large number of patients affected by HVL during the study period who had to travel to other mesoregions. The state capital located in the Belém Metropolitan Region, is the main point of attraction, as it has the most qualified physical and professional infrastructure [40,58,59,60,61]. This convergence with cross-sectional flow poses a major problem for the Unified Health System in terms of regulating and treating cases, which points to the need for decentralizing the services for dealing with the disease. Moreover, this situation creates additional difficulties related to the disease itself, due to the long journey that patients have to make, the prolonged hospitalizations far from their families, and the psychological, social and economic implications involved. These facts starkly reveal the inequities related to the unequal risk of becoming ill and dying in the Amazon [58,59,60,61].

With regard to the distances patients traveled, this variable had a direct influence on access to health services, with a greater possibility of access associated with the existence of highways and waterways. In this regard, even considering that the vast majority of clinical and laboratory diagnoses with less specificity for the disease occurred in the patient’s municipality of origin, the spatial analysis showed their geographical segregation in terms of access to health services. This fact highlights a major challenge for those who need TAFH because they live more than 150 km from the place of care [62], especially in a state that has an extensive territorial dimension, several population groups residing on islands, floodplain, mangroves and dry land, huge social inequalities, low coverage of health services, a quantitative and qualitative shortage of professionals, delays in laboratory confirmation of the disease and a lack of diagnostic resolution [39,58,59,60,61].

The Kernel technique showed that the distribution of HVL was not homogeneous in the study mesoregions. The high and very high densities of cases of the disease observed in municipalities located near rivers and highways including the formation of epidemiological corridors, suggests that these routes are important means of vectoring the disease, due to migratory processes that involve the circulation of humans and domestic animals without zoonotic control [32,63]. This health scenario has been present throughout the socio-spatial formation of the state of Pará, which initially took place along the Amazonian rivers, forming the riverside communities, and later along the highway, in the context of national integration [64]. These conditions confirm that HVL health surveillance actions must go beyond the border demarcations of the mesoregions, since the municipalities are historically interconnected, and their economic dynamics are totally associated with these road and waterway networks.

The spatial analysis of the types of use and cover of land showed that the areas where the highest levels of environmental change have occurred also have the highest numbers of cases of HVL. Within this context the southeastern mesoregion of Para, which plays an important role in the Pará economy and reported 1376 cases of the disease, is one of the most critical regions in the Amazon in terms of changes in vegetation cover. This mesoregion has large mineral reserves and water potential in its territory that have been a magnet for large development projects managed by national and international conglomerates, as well as presenting an established scenario of the disease, showing a process that reflects the inequality of the distributive structure based on the polarization of wealth, the spatial segregation of poverty and socioeconomic and territorial disparities [21,32,53].

The Northeast Pará mesoregion also has a history of high rates of deforestation, conversion of forest into pasture and 1070 cases of the disease. The territory is currently undergoing a major expansion of oil palm cultivation and mineral extraction, which has led to major changes in its land, socio-economic and environmental structure, especially in the micro-regions of Tomé-Açu and Cametá, which have municipalities with very high and high prevalence of the disease [29,30,31,32,33,65]. As for the Belém Metropolitan mesoregion, deforestation has occurred as a result of the urban occupation process, especially in Belém and Ananindeua, which are among the ten worst municipalities in Brazil in terms of sanitation and reported a large number of cases during the study period [61,66].

In the Marajó mesoregion, changes in vegetation cover have been influenced by activities linked to livestock farming, extractive activities and agriculture. Twelve of the municipalities in this region are part of the Marajó Environmental Protection Area, which has areas of tropical forest and great socio-biodiversity and a majority of its population made up of African and Indigenous ethnic groups. It is one of the poorest regions in Brazil and the low number of cases of HVL reported may be related to the precarious coverage of health services, the shortage of professionals and the operational difficulties encountered in diagnosing and reporting this disease in the primary health care network [67].

The occurrence of cases of HVL in Conservation Units and Indigenous Lands in the northeast and southeast mesoregions of Pará encourages reflection regarding the exogenous, hierarchical and Eurocentric model of occupation and economic exploitation of these regions, which treats the different socio-environmental realities homogeneously and unsustainably uses these territories as an inexhaustible source of raw materials that exposes local and indigenous populations to the risk factors for the disease. These epidemiological scenarios are indicative of the synergistic and cumulative impacts related to the pressure of socio-economic dynamics that consider humans and the environment as exclusionary poles, determining conflicts of multiple interests in land use and occupation between the social agents who live in these parts of the Eastern Amazon [32,68].

In short, the direct spatial correlation (weak and strong) between areas with significant numbers of HVL and areas with deforestation and human impacts indicates that the development model implemented in these Amazonian mesoregions has produced patterns of landscape alteration that involve different stages of ecological succession of the vegetation and have disregarded the possible impacts on the relationship between health and the environment. This is especially the case with regard to vegetation removal, changes in the population’s way of life, and the expansion of ecological niches for vectors and reservoirs of the disease [13,14,15,16,50,69,70,71,72]. With this in mind, when analyzing the impacts of deforestation on diseases in the Amazon Scarro [13], highlighted the need to include the incidence of leishmaniasis as a variable in the social costs for the environment in licensing projects that involve the suppression of vegetation, since deforesting 1% of a given area can increase the incidence of disease cases by 5% to 9%.

The complex relationships of the different epidemiological scenarios observed in this research reveal a fundamental element that must be considered when planning public health policies and that is related to understanding the disease beyond its biological causes. In other words, one must understand HVL from the perspective that social subjects are in constant socio-environmental, behavioral, cultural, economic and political interactions. And, as shapers of space, they directly influence the cohabitation and coexistence of different species in the Amazonian ecosystems, including vectors and hosts of diseases such as HVL.

## 5. Conclusions

This study analyzed the spatial distribution of HVL and its relationship with socioeconomic, environmental and public health policy variables in four mesoregions of the state of Pará. The epidemiological profile found is recurrent in other areas of the Amazon. The occurrence of high densities of HVL, high socioeconomic indicators with disordered land use and coverage in the mesoregions showed that these territories are socially and economically structured around the unsustainable use of natural resources. That has favored the social and environmental production of the disease, including in conservation units and Indigenous lands, with this socioeconomic dynamic being the antithesis of sustainable development.

We conclude that there is a need to develop and implement more efficient and effective public policies aimed at mitigating the complex socio-epidemiological scenario of HVL in the study mesoregions. These include improving housing, health and basic sanitation conditions, developing inclusive, permanent, systematic and multi-level health education measures aimed at promoting One Health in all its dimensions, as well as implementing economic practices that prioritize social equity and respect for environmental limits in these territories.

## Figures and Tables

**Table 1:** Epidemiological profile of Human Visceral Leishmaniasis in the metropolitan mesoregions of Belém, Marajó, Northeast Pará and Southeast Pará, in the period 2011–2022.

Variable	Marajó	Metropolitan Belém	Northeast Pará	Southeast Pará	Total
	*n* (%)	*n* (%)	*n* (%)	*n* (%)	*n* (%)
Gender					
Male	30 (52.6)	116 (63.7)	614 (57.4)	848 (61.6)	1608 (59.9)
Female	27 (47.4)	66 (36.3)	456 (42.6)	528 (38.4)	1077 (40.1)
*p*-value ^1^	0.7911	0.0003	<0.0001	<0.0001	<0.0001
Age Group					
Children (0–12 years old)	36 (63.2)	91 (50.0)	720 (67.3)	679 (49.3)	1526 (56.8)
Adolescents (13–18 years old)	3 (5.3)	20 (11.0)	68 (6.4)	96 (7.0)	187 (7.0)
Adults (19–59 years old)	16 (28.1)	64 (35.2)	252 (23.6)	532 (38.7)	864 (32.2)
Elderly (≥60 years old)	2 (3.5)	7 (3.8)	30 (2.8)	69 (5.0)	108 (4.0)
*p*-value ^1^	<0.0001	<0.0001	<0.0001	<0.0001	<0.0001
Ethnic Groups					
Brown Skin	42 (73.7)	149 (81.9)	833 (77.9)	1009 (73.3)	2033 (75.7)
Black	10 (17.5)	9 (4.9)	89 (8.3)	133 (9.7)	241 (9.0)
White	2 (3.5)	13 (7.1)	60 (5.6)	147 (10.7)	222 (8.3)
Yellow	3 (5.3)	10 (5.5)	6 (0.6)	8 (0.6)	17 (0.6)
Indigenous	0 (0.0)	1 (0.5)	4 (0.4)	8 (0.6)	13 (0.5)
Unknown	0 (0.0)	0 (0.0)	78 (7.3)	71 (5.2)	159 (5.9)
*p*-value ^1^	<0.0001	<0.0001	<0.0001	<0.0001	<0.0001
Schooling					
Does not apply ^2^	31 (54.4)	69 (37.9)	606 (56.6)	543 (39.5)	1249 (46.5)
Illiterate	0 (0.0)	2 (1.1)	23 (2.1)	38 (2.8)	63 (2.3)
Primary school	9 (15.8)	58 (31.9)	283 (26.4)	376 (27.3)	726 (27.0)
High school	6 (10.5)	11 (6.0)	38 (3.6)	117 (8.5)	172 (6.4)
Superior	0 (0.0)	0 (0.0)	2 (0.2)	10 (0.7)	12 (0.4)
Unknown	11 (19.3)	42 (23.1)	118 (11.0)	292 (21.2)	463 (17.2)
*p*-value ^1^	<0.0001	<0.0001	<0.0001	<0.0001	<0.0001
Zona					
Rural	40 (70.2)	97 (53.3)	775 (72.4)	157 (11.4)	1069 (39.8)
Urban	15 (26.3)	74 (40.7)	209 (19.5)	1198 (87.1)	1496 (55.7)
Periurban	2 (3.5)	2 (1.1)	19 (1.8)	2 (0.1)	25 (0.9)
Unknown	0 (0.0)	9 (4.9)	67 (6.3)	19 (1.4)	95 (3.5)
*p*-value ^1^	<0.0001	<0.0001	<0.0001	<0.0001	<0.0001
Total Cases	57 (2.1)	182 (6.8)	1070 (39.9)	1376 (51.2)	2685 (100.0)

n = Number of cases; ^1^ *p* < 0.05 (chi-square, adherence); ^2^ Children below school age.

## Data Availability

The data used in this study regarding the socioeconomic and environmental variables can be found at https://cidades.ibge.gov.br/ (accessed on 5 December 2023) and https://brasil.mapbiomas.org (accessed on 4 March 2024). All figures in this paper used public data and were prepared by the authors.
